# Neutrophil-Derived Myeloperoxidase Facilitates Both the Induction and Elicitation Phases of Contact Hypersensitivity

**DOI:** 10.3389/fimmu.2020.608871

**Published:** 2021-01-25

**Authors:** Anna Strzepa, Cody J. Gurski, Landon J. Dittel, Marian Szczepanik, Kirkwood A. Pritchard, Bonnie N. Dittel

**Affiliations:** ^1^ Versiti Blood Research Institute, Milwaukee, WI, United States; ^2^ Department of Medical Biology, Faculty of Health Sciences, Jagiellonian University Medical College, Krakow, Poland; ^3^ Department of Neurology, Medical College of Wisconsin, Milwaukee, WI, United States; ^4^ Department of Surgery, Division of Pediatric Surgery, Medical College of Wisconsin, Milwaukee, WI, United States; ^5^ Deparment of Microbiology and Immunology, Medical College of Wisconsin, Milwaukee, WI, United States

**Keywords:** contact hypersensitivity, interleukin 1β, myeloperoxidase, neutrophil, vascular permeability

## Abstract

**Background:**

Allergic contact dermatitis (ACD) is a common skin disorder affecting an estimated 15-20% of the general population. The mouse model of ACD is contact hypersensitivity (CHS), which consists of two phases: induction and elicitation. Although neutrophils are required for both CHS disease phases their mechanisms of action are poorly understood. Neutrophils release myeloperoxidase (MPO) that through oxidation of biomolecules leads to cellular damage.

**Objectives:**

This study investigated mechanisms whereby MPO contributes to CHS pathogenesis.

**Methods:**

CHS was induced in mice using oxazolone (OX) as the initiating hapten applied to the skin. After 7 days, CHS was elicited by application of OX to the ear and disease severity was measured by ear thickness and vascular permeability in the ear. The role of MPO in the two phases of CHS was determined utilizing MPO-deficient mice and a specific MPO inhibitor.

**Results:**

During the CHS induction phase MPO-deficiency lead to a reduction in IL-1β production in the skin and a subsequent reduction in migratory dendritic cells (DC) and effector T cells in the draining lymph node. During the elicitation phase, inhibition of MPO significantly reduced both ear swelling and vascular permeability.

**Conclusion:**

MPO plays dual roles in CHS pathogenesis. In the initiation phase MPO promotes IL-1β production in the skin and activation of migratory DC that promote effector T cell priming. In the elicitation phase MPO drives vascular permeability contributing to inflammation. These results indicate that MPO it could be a potential therapeutic target for the treatment of ACD in humans.

## Highlights

- Contact hypersensitivity induction in myeloperoxidase-deficient mice is characterized by lower IL-1β in skin resulting in reduced migration of dendritic cells to the draining lymph node and subsequented attenuated effector T cell priming.
- In the elicitation phase of contact hypersensitivity myeloperoxidase is a critical mediator driving vascular permeability.

## Introduction

Allergic contact dermatitis (ACD) arises after multiple non-symptomatic exposures to low molecular weight substances called haptens affecting 20% of the general population (1, 2). The mouse model of ACD is contact hypersensitivity (CHS), which is a T cell-dependent immune response classified as a type four hypersensitivity reaction or delayed hypersensitivity response (DTH). CHS consists of two consecutive phases, induction and elicitation (3–5). For induction, hapten is applied to bare skin, which leads to activation of keratinocytes and mast cells and subsequent neutrophil recruitment (3, 4). Innate immune cell activation in the skin was shown to lead to elevated levels of interleukin (IL-1β), which contributed to dendritic cell (DC) activation and migration to local lymph nodes where they induced antigen-specific T cell differentiation towards the Th1/Th17 phenotype (6–9). During the elicitation phase, hapten is applied to the ears leading to activation of local keratinocytes and mast cells and subsequent neutrophil migration into the site (10, 11). Increased vascular permeability leads to ear swelling, which facilitates migration of antigen-specific Th1/Th17 cells into the site (4, 6, 9).

Neutrophils were shown to be crucial for both the induction and elicitation phases of CHS (12, 13). The most abundantly expressed protein in neutrophils is myeloperoxidase (MPO), which utilizes hydrogen peroxide to create hypochlorous acid that, in turn, oxidizes essential biomolecules leading to cell dysfunction and death (14–16). In addition, MPO and its products have been shown to regulate the immune response in variable ways including limiting DC activation (17) and increasing the production of proinflammatory cytokines (15, 18, 19). The usage of MPO-deficient mice and MPO inhibitors has provided evidence for MPO being pathogenic in a variety of diseases (15, 20, 21).

We investigated the role of MPO in CHS induction and elicitation by utilizing active and adoptive transfer models of oxazolone (OX) CHS incorporating MPO deficiency and inhibition. We showed that MPO promoted the induction of adaptive immune responses by promoting IL-1β production thereby leading to DC activation and migration from skin to draining lymph nodes and subsequent T cell priming. In the elicitation phase, MPO was shown to drive vascular permeability contributing to local inflammation. Our data indicate that MPO could be a potential therapeutic target for ACD in humans.

## Methods

### Mice

C57BL/6 and B6.129X1-Mpotm1Lus/J (*Mpo^−/−^*) mice were purchased from The Jackson Laboratory (Bar Harbor, ME) and housed under specific pathogen-free conditions in the Translational Biomedical Research Center, Medical College of Wisconsin. All experiments were conducted with Institutional Animal Care and Use Committee approval.

### Reagents and Antibodies

The MPO inhibitor tripeptide N-acetyl lysyltyrosylcysteine amide (KYC) was synthesized by the Protein Core Laboratory of Versiti Blood Research Institute. 10x HBSS, 1x HBSS, Dibutyl phthalate, Evans Blue, FITC (Isomer I), Formamide, OX, and Percoll were purchased from Sigma-Aldrich (St. Louis, MO). Antibodies are described in **Table S1**.

### Flow Cytometry

Axillary and inguinal lymph nodes were isolated and single cell suspensions prepared. Treg were detected with the mouse Foxp3/Transcription Factor Staining Buffer Set (ThermoFisher Scientific, Waltham, MA). IFN-γ was detected by intracellular cytokine staining using the Intracellular Fixation & Permeabilization Buffer Set (ThermoFisher Scientific, Waltham, MA) (22). Samples were collected on a FACS Aria flow cytometer (BD Biosciences, San Jose, CA) and the data were analyzed using FlowJo (Tree Star, Ashland, OR).

### CHS Induction and KYC Treatment

Mice were sensitized with 0.15 ml of 3% OX in acetone-ethanol (1:3) on the shaved abdomen and chest (3). Control mice were sham sensitized with acetone-ethanol alone. Four d later, basal ear thickness was measured with a micrometer (Mitutoyo, Tokyo, Japan), followed by challenge of on both sides of the ears with 1% OX (10 µl) in olive oil-acetone (1:1). Twenty-four h after challenge ear thickness was measured. Basal ear thickness was subtracted from respective values obtained 24 h after challenge. Ear swelling was expressed as µm ± SE. For some experiments, groups of mice were i.p. injected with either PBS or KYC (0.03 mg/kg) twice daily starting 12 h before sensitization.

### Neutrophil Depletion and T effector (Teff) Adoptive Cell Transfer

For adoptive cell transfer, donor C57BL/6 and *Mpo^−/−^* mice were sensitized with 0.15 ml 3% OX in acetone-ethanol mixture (1:3). Four d later, splenocyte suspensions were depleted of neutrophils with anti-Ly6G-biotin using the Biotin Selection Kit Easy Step from Stem Cell Technologies (Vancouver, BC, Canada). Depletion efficiency was >99%. 5 x 10^7^ *Mpo^-/-^* Teff cells were i.v. transferred to naïve WT recipients and 5 x 10^7^ WT Teff were transferred to naïve WT and *Mpo^-/-^* recipients. After 2 h, 1% OX in olive oil-acetone (1:1) was applied to the ears and ear thickness was measured 24 h later.

### Evans Blue Permeability Assay

To evaluate vascular permeability, mice were sham or 3% OX sensitized followed by ear challenge with 1% OX four d later and 23 h later the mice were i.v. injected with 1% Evans blue (83 µg/g body weight) and after 1 h a 6 mm diameter ear punch biopsy was collected and placed into 0.5 ml of formamide. The ear tissue was incubated for 18 h at 37°C and after centrifugation, the optical density (OD) of Evens blue in the supernatant was read at 565 nm and a standard curve was used to determine the EB concentration.

### Neutrophil Isolation

Neutrophils were isolated at room temperature from bone marrow (BM) extracted from the femurs and tibiae of WT mice. Total BM cells were resuspended in 45% Percoll that layered on a Percoll gradient of 66%, 60%, 55%, and 50%. The cells were centrifuged at 2800 rpm for 30 min without using the break. The bottom band of cells between the 60% and 66% layers were collected, resuspended in 10 ml HBSS with 0.1% BSA, centrifuged at 1200 rpm for 10 min and washed twice with HBSS. Cell purity was determined by flow cytometry and 96% of the CD11b^+^ cells were neutrophils (CD11b^+^Ly6C^+^Gr1^+^).

### Electric Cell-Substrate Impedance Sensing

Using the ECIS Z System (Applied BioPhysics, Inc. Troy NY) a 8W10E^+^ array was stabilized and coated with 200 µl of 0.1% gelatin for 30 min at 37° C prior to seeding with bEnd3 cell line cells at 80,000 per well. The cells were cultured until capacitance (nF) at 64,000 Hz reached the range from 1 to 5nF. For the co-culture assay the growth medium was exchanged with HBSS (Ca^2+^, Mg^2+^) with 5.5 mM glucose. 0.2 x 10^6^ purified neutrophils were stimulated with 2 μM PMA for 12 min in HBSS. Activated neutrophils were treated with 25 μM KYC for 10 min. Neutrophils were added to the endothelial monolayer and impedance was collected at 4000 Hz every 15 s for up to 4.5 h.

### FITC-Induced Cutaneous DC Migration Assay

To evaluate the migration of DC from skin to draining lymph nodes, the shaved abdomen and chest were painted with 100 μl of 0.5% FITC isomer I dissolved in a 1:1 (v/v) acetone/dibutyl phthalate mixture (23). Six h\ later, draining lymph nodes were collected and the absolute number of FITC^+^ DC and their percentage among B220^-^FITC^+^ cells was determined by flow cytometry.

### ELISA

To evaluate IL-1β concentration in sensitized skin, WT and *Mpo^-/-^* were sensitized with 0.15 ml of 3% OX in acetone-ethanol (1:3) on the shaved abdomen and chest. Control mice were sham sensitized with acetone-ethanol alone. After 6 h, abdominal skin was excised and 6 mm biopsy punches were collected to 200 μl of modified RIPA buffer (10 mM Tris-HCL ph=7.4, 150 mM NaCl, 0.25%% Triton X) with Protease Inhibitor Cocktail (Sigma-Aldrich, St. Louis, MO). The samples were homogenized and the supernatants were transferred to a new tube and the beads were washed with 50 μl buffer, followed by centrifugation (15 min, 4° C, 14000 × g). The IL-1β concentration was determined using the Mouse IL-1β/IL-1F2 DuoSet ELISA (R&D Systems, Minneapolis, MN) according to manufacture instructions.

### Statistical Analysis

Normally distributed data were analyzed using ANOVA followed by Bonferroni’s Multiple Comparison Test for comparison between multiple groups. For two-group comparison, the unpaired t-test was used. Data are presented as mean values ± SEM and a p-value ≤0.05 was considered significant using GraphPad Prism (San Diego, CA).

## Results

### Attenuation of the CHS Response in MPO-Deficient Mice

**Figure 1:**
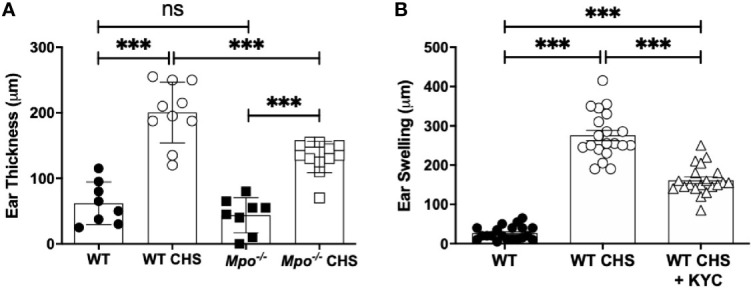
MPO-deficiency and inhibition reduced inflammation in the OX CHS model. Mice were sham (WT, *Mpo^-/-^*) or OX (3%) sensitized and four d later were ear challenged with OX (1%). **(A)** Ear thickness was measured with a caliper 24 h later. **(B)** Mice were i.v. administered 0.03 mg/kg KYC every 12 h starting at -12 h prior to sensitization until the end of the study. Each symbol represents one ear with data from three separate experiments shown. ***p≤0.001, ns, not significant.

Here, we investigated MPO as an neutrophil effector molecule in CHS using ear swelling to quantitate disease penetrance. There was no difference in ear thickness between WT and *Mpo^-/-^* mice following sham sensitization and OX challenge to the ear (**Figure 1A**). However, following sensitization with OX, WT mice exhibited a significant increase in ear thickness 24 h after OX challenge (**Figure 1A**). Although ear swelling increased in *Mpo^-/-^* mice following sensitization and challenge, it was significantly lower than in WT mice (**Figure 1A**).

To confirm a role for MPO in the pathogenesis of CHS, we utilized KYC, a specific inhibitor of MPO toxic oxidant production that we developed (24). KYC (0.03 mg/kg) was i.v. administered every 12 h starting at -12 h. Following OX challenge, MPO inhibition with KYC significantly reduced, but did not prevent ear swelling (**Figure 1B**). The identical result was obtained in mice in which KYC administration was initiated 1 day following sensitization (data not shown). The optimal dosage of KYC (0.03 mg/kg) was experimentally determined in a dose-response study and was consistent with our previous studies (25, 26).

### MPO Enhances the Induction of the Adaptive Immune Response in CHS

**Figure 2:**
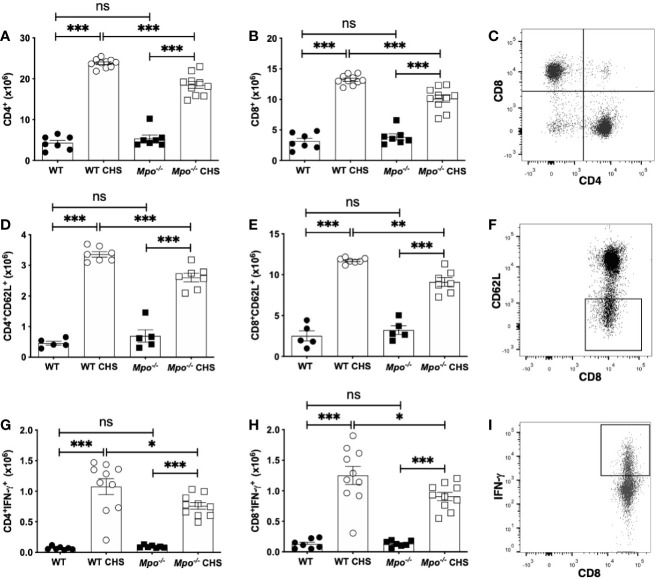
MPO-deficiency reduces the induction of the adaptive immune response. WT and *Mpo^-/-^* mice were OX (3%) or sham immunized. After four d lymph nodes cells were collected, and single cell suspensions were stained with fluorochrome-conjugated antibodies and analyzed by flow cytometry and the absolute cell number of CD4^+^ T cells **(A)**, CD8^+^ T cells **(B)**, CD4^+^CD62L^-^ T cells **(D)**, CD8^+^CD62L^-^ T cells **(E)**, CD4^+^IFN-γ^+^ T cells **(G)**, and CD8^+^IFN-γ^+^ T cells **(H)** was determined. Representative flow cytometry gating is shown for CD3-gated CD4 and CD8 T cells **(C)**, CD8^+^CD62L^-^ T cells **(F)**, and CD8^+^IFN-γ^+^ T cells **(I)**. Each symbol represents data from one mouse with data from two to three independent experiments shown. *p≤0.05; **p≤0.01; ***p≤0.001; ns, not significant.

The reduction in ear swelling in *Mpo^-/-^* (**Figure 1A**) and KYC-treated (**Figure 1B**) mice indicated that MPO could potentially promote efficient T cell priming. Thus, to establish the impact of MPO on the induction of the adaptive immune response to OX, we quantitated T cell subsets in the skin draining lymph nodes four d after sensitization. We found no difference in the absolute number of CD4 (**Figure 2A**) or CD8 T cells (**Figure 2B**) in WT as compared to *Mpo^-/-^* mice that were sham sensitized. In contrast, there as a significant reduction in the absolute number of both CD4 (**Figure 2A**) and CD8 (**Figure 2B**) T cells in *Mpo^-/-^* OX sensitized mice. A representative flow cytometry plot illustrating the gating strategy for all groups is shown in **Figure 2C**. Because there was a significant decrease in the absolute number of CD4 and CD8 T cells in *Mpo^-/-^* CHS mice, we next determined whether this was due to an attenuation of T cell priming. First, we examined CD62L, a marker of naïve T cells that is shed upon their activation (27). In both CD4 (**Figure 2D**) and CD8 (**Figure 2E**) Teff cells a significant decrease in CD62L^-^ cells was observed in sensitized *Mpo^-/-^* mice as compared to WT mice. A representative flow cytometry gating strategy for all groups is shown in **Figure 2F**. Similar results were obtained when IFN-γ producing CD4 and CD8 Teff were quantitated (**Figures 2G–I**). These results suggest that MPO affects the magnitude of the adaptive immune response, but does not alter the nature of naïve T cell priming.

### MPO-Deficiency Does Not Impact Lymphocyte Proliferation

**Figure 3:**
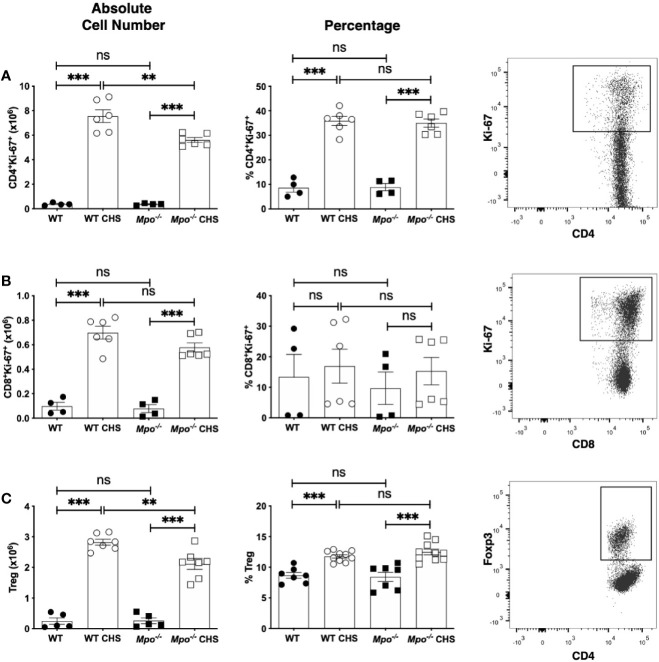
MPO-deficiency does not influence CD4 and CD8 T cell proliferation or Treg numbers in the draining lymph node during induction of CHS. WT and *Mpo^-/-^* mice were OX (3%) or sham immunized. After four d lymph nodes cells were collected, and single cell suspensions were stained with fluorochrome-conjugated antibodies and analyzed by flow cytometry. The absolute cell number and frequency of Ki-67^+^TCRβ^+^CD4^+^ T cells **(A)**, Ki-67^+^TCRβ^+^CD8^+^ T cells **(B)** and TCRβ^+^CD4^+^Foxp3^+^ Treg **(C)** was determined by flow cytometry. Each symbol represents data from one mouse with data from two to three separate experiments shown. Representative flow cytometry gating for each cell type is shown in the right column **(A–C)**. **p≤0.01; ***p≤0.001; ns, not significant.

To determine whether the reduction in Teff cells was due to reduced proliferation, we quantitated the number and percentage of Ki-67^+^ T cells (28). For both CD4 (**Figure 3A**) and CD8 (**Figure 3B**) T cells there was no significant difference in the absolute number or percentage of Ki-67^+^ cells between WT and *Mpo^-/-^* sham sensitized mice. While there was a significant decrease in the absolute number of Ki-67^+^ CD4 T cells in *Mpo^-/-^* mice as compared to WT mice OX sensitized mice, the percentage of cells undergoing proliferation was not different (**Figure 3A**). For CD8 T cells, there was not a significant difference in the absolute number nor percentage of cells undergoing proliferation (**Figure 3B**). Since the extent of the adaptive immune response is controlled by CD4^+^Foxp3^+^ T regulatory cells (29–31), we next determined whether their numbers were increased in *Mpo^-/-^* mice. The absolute number and percentage of Treg was not different between sham sensitized WT and *Mpo^-/-^* mice (**Figure 3C**). In sensitized mice, the absolute number of Treg was reduced in *Mpo^-/-^* mice without a difference in the percentage of Treg (**Figure 3C**). Representative flow cytometry gating strategies for all groups are shown (**Figures 3A–C**). These cumulative data indicate that the reduced adaptive immune response in *Mpo^-/-^* mice is not due to changes in the level of CD4 and CD8 T cell proliferation or increased Treg numbers.

### MPO Enhances DC Migration to the Draining Lymph Nodes

**Figure 4:**
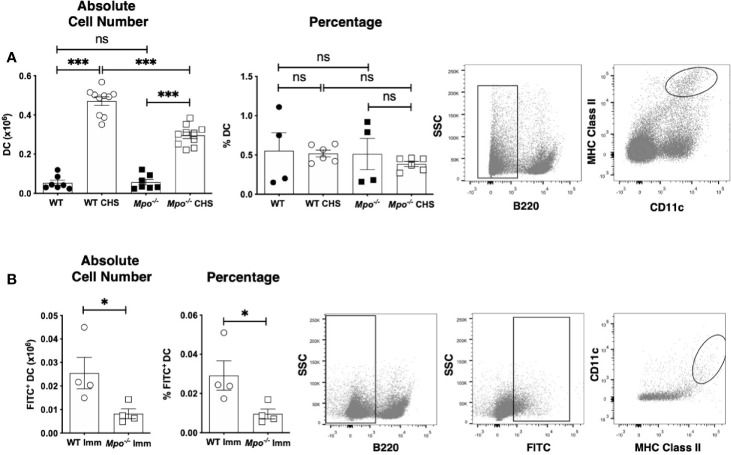
MPO-deficiency reduces DC migration from skin to the draining lymph nodes during CHS. WT and *Mpo^-/-^* mice were OX (3%) or sham immunized. After 4 days, skin-draining auxiliary lymph nodes were collected, and single cell suspensions were stained with fluorochrome-conjugated antibodies and analyzed by flow cytometry and the absolute cell number and percentage of B220^-^CD11c^+^ MHC class II^+^ DC **(A)** was determined. WT and *Mpo^-/-^* mice were skin painted with 0.5% FITC and 6 h later the absolute number and percentage of FITC^+^ DC among B220^-^FITC^+^ cells was determined **(B)**. Each symbol represents data from one mouse with data from three **(A)** separate experiments shown or is one representative experiment of two **(B)**. Representative flow cytometry gating is shown on the right for each analysis. **(A, B)**. *p≤0.05; ***p≤0.001; ns, not significant.

Because MPO-deficiency led to a decrease in the number of primed T cells in the draining lymph node in CHS, we next determined whether this was correlated to a similar reduction in DC cell numbers. When we quantitated the absolute number of migratory DC (B220^-^CD11c^+^MHCII^+^) (23) in the draining lymph nodes four d after OX sensitization, we found that *Mpo^-/-^* mice had a significant reduction in the absolute number of DC, but the percentage of DC was not changed (**Figure 4A**). This finding was not due to reduced numbers or the percentage of migratory DC in the sham control mice (**Figure 4A**). To confirm that MPO-deficiency specifically impacted migratory DC, mice were skin painted with FITC and the number of FITC^+^ DC in the draining lymph node was quantitated 24 h later. In the absence of FITC skin painting, FITC^+^ migratory DC were not detectable. In FITC painted mice, there was a significant decrease in the absolute number and percentage of FITC^+^ DC (**Figure 4B**). Representative flow cytometry gating for all groups is shown (**Figures 4A, B**). These data suggest that T cell priming is reduced in *Mpo^-/-^* mice as the result of fewer skin DC migrating to the draining lymph node. Representative flow cytometry gating is shown (**Figures 4A, B**).

### MPO Deficiency Hampers IL-1β Production in the Skin in Response to OX Sensitization

**Figure 5:**
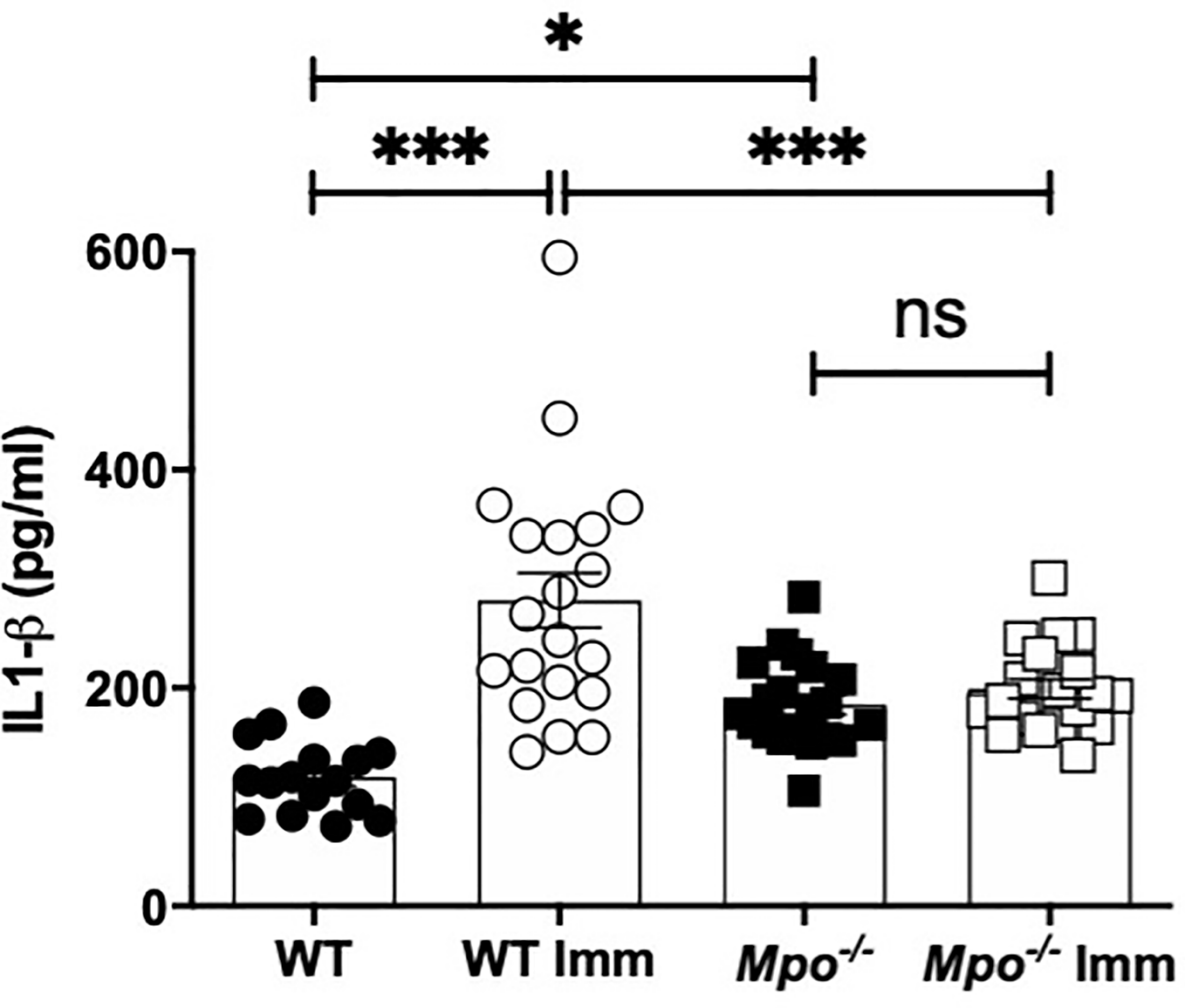
MPO-deficiency reduces IL-1β levels in skin biopsies. WT and *Mpo^-/-^* mice were OX (3%) or sham immunized. After 6 h skin biopsies were collected and IL-1β levels were quantitated by ELISA. Each symbol represents data from one mouse with data from two separate experiments shown. *p≤0.05; ***p≤0.001; ns, not significant.

To investigate the mechanism of reduced migratory DC in the draining lymph nodes IL-1β production in the skin was measured because of its role in promoting DC migration (7, 8). Following sham sensitization, IL-β in the skin was significantly higher in *Mpo^-/-^* mice as compared to WT (**Figure 5**). Six h following OX challenge in sensitized mice the level of IL-1β in the skin significantly increased in WT, but was unchanged in *Mpo^-/-^* mice (**Figure 5**). These data indicate that MPO is essential for the generation of active IL-1β expression in the skin that facilitates the activation and subsequent migration of DC from the skin to the draining lymph node to initiate T cell priming to the sensitizing hapten.

### Attenuation of the CHS Response in MPO-Deficient Mice

**Figure 6:**
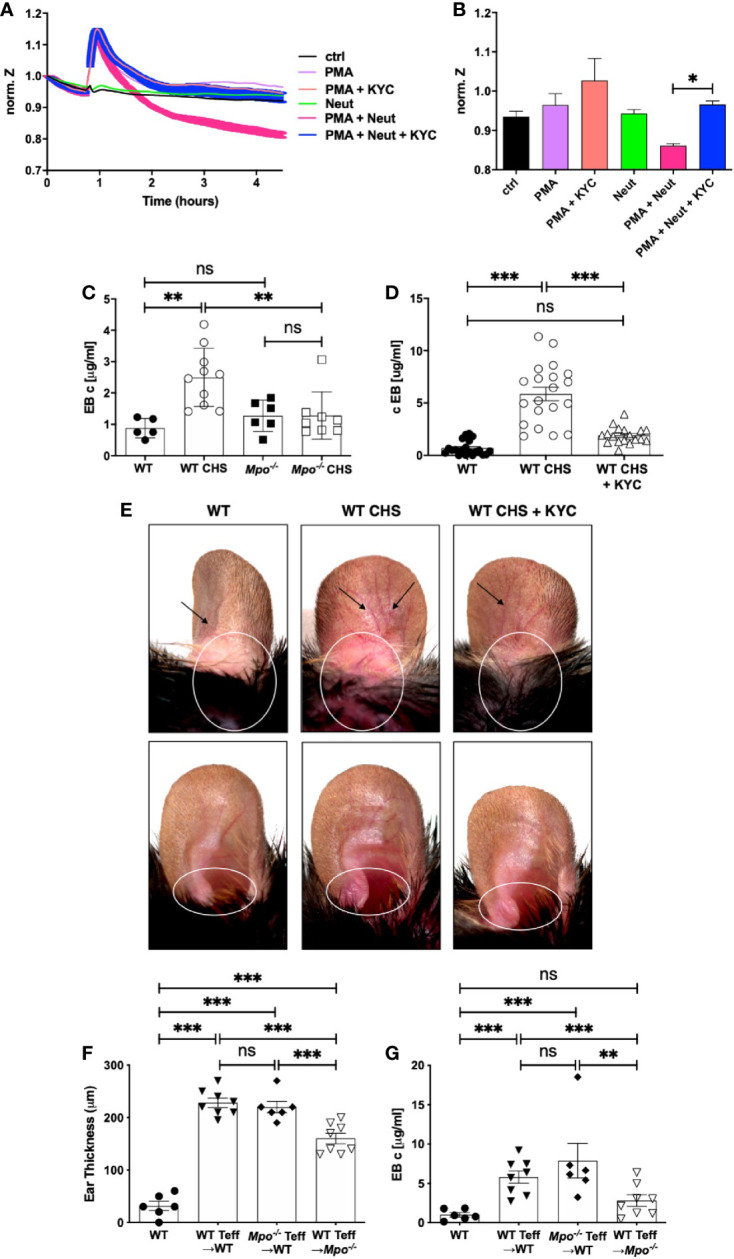
MPO supports the elicitation of CHS by increasing vascular permeability. **(A, B)** Confluent monolayers of bEnd3 cells were established on collagen-coated gold electrodes and impendence measurements at 4 kHz were conducted. **(A)** After establishment of the steady state level of impendence, impendence was measured in medium alone (control) or in the presence of unstimulated neutrophils (neut), PMA (20 μM), PMA + KYC (25 μM), PMA + neutrophils, or PMA + neutrophils + KYC. **(B)** At the 2.5 h time point impendence level was quantitated for each condition. The experiment shown is representative of two. C-E) Mice were sham (WT, *Mpo^-/-^*) or OX (3%) sensitized and 4 days later were ear challenged with OX (1%). Vascular permeability was measured 24 h later by Evans blue leakage from the vascular into the tissue 60 min after i.v. administration **(C, D)**. **(D, E)** Mice were i.v. administered 0.03 mg/kg KYC every 12 h starting at -12 h prior to sensitization until the end of the study. Each symbol represents one ear with data from three separate experiments shown. **(E)** Twenty-four hours after OX challenge mouse ears were photographed using Zeiss Lumar.V12 stereoscope at 6.4x. Blood vessels are indicated by arrows and areas exhibiting inflammation are circled. The top panels show the dorsal side and the bottom panels the ventral side of the ear. **(F, G)** WT or *Mpo^-/-^* mice were OX (3%) immunized and four d later Teff cells were isolated and depleted of neutrophils and adoptively transferred into WT or *Mpo^-/-^* recipients that were subsequently ear challenged with OX (1%). Twenty-four h later ear thickness **(F)** and vascular permeability **(G)** was measured. Each symbol represents one ear with data from one representative experiment of two shown. *p≤0.05; **p≤0.01; ***p≤0.001; ns, not significant.

Here, we investigated MPO as an neutrophil effector molecule in the CHS elicitation as a driver of vascular permeability. This question was first addressed using ECIS, which is an *in vitro* system to measure cell behavior of adherent cell layers (32). ECIS was used to quantitate neutrophil-derived MPO disruption of an endothelial cell monolayer using bEnd3 cells (33–35). Electric current was applied and following equilibration, there was no change in the electrical potential for greater than four h in medium or after addition of resting neutrophils (**Figure 6A**). In contrast, neutrophils activated with PMA induced a sharp increase in impedance, that returned to baseline after ~1 h and then steadily decreased over two h (**Figure 6A**). The drop below baseline indicated a loss of barrier function. Inclusion of KYC with the PMA activated neutrophils completely inhibited the loss of barrier function (**Figure 6A**). The spike in increased barrier integrity was evident in every sample containing PMA (**Figure 6A**). Quantitation of the 2.5 h timepoint showed a significant reduction in impedance when KYC was added to PMA activated neutrophils (**Figure 6B**).

We next determined whether MPO contributes to vascular permeability in CHS by measuring vascular leakage with Evans blue. In sham sensitized mice, there was no difference in vascular permeability between WT and *Mpo^-/-^* mice (**Figure 6C**). Sensitized WT mice exhibited a significant increase in vascular leakage, while *Mpo^-/-^* mice did not (**Figure 6C**). To confirm a role for MPO, we treated mice with KYC every 12 h from day -0.5, which led to a significant inhibition of vascular leakage (**Figure 6D**). In **Figure 6E**, images are shown of mouse ears that were sham sensitized followed by OX challenge on the ear 4 d later that exhibit no obvious inflammation as compared to mice receiving both OX sensitization and challenge, which exhibited dilated blood vessels (arrows) and evidence of inflammation as indicated by increased redness (circle) (**Figure 6E**). Inflammation in mice treated with KYC to inhibit MPO was reduced as shown by decreased blood vessel dilation and redness (**Figure 6E**).

To confirm the critical role for neutrophil-derived MPO in the elicitation phase of CHS, we performed an adoptive transfer experiment in which OX primed Teff cells from WT or *Mpo^-/-^* donors were depleted of neutrophils prior to transfer into naïve recipients, which were subsequently ear challenged with OX. Teff cells from both WT and *Mpo^-/-^* mice transferred into WT donors induced similar levels of ear swelling (**Figure 6F**) and vascular permeability (**Figure 6G**). In contrast, when neutrophil depleted Teff from WT mice were transferred into *Mpo^-/-^* recipients, there was a significant decrease the level of ear swelling (**Figure 6F**) with no accompanied vascular permeability (**Figure 6G**). These data indicate that neutrophils drive the elicitation phase by release of MPO that promotes vascular permeability.

## Discussion

**Figure 7:**
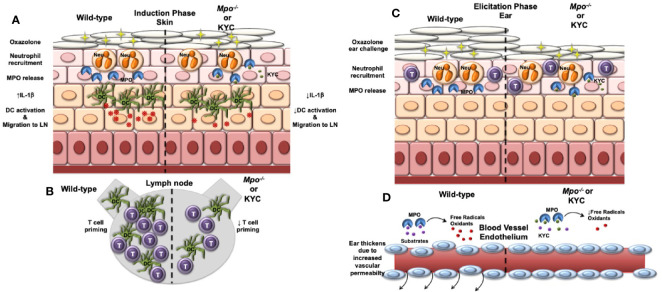
Model outlining the role of MPO in the induction and elicitation phases of CHS. **(A)** For induction of CHS oxazolone (OX) is applied to the skin, which results in migration of neutrophils into the skin and release of MPO and subsequent induction of IL-1β production within the skin. Skin resident dendritic cells (DC) take up Ox and become activated that is facilitated by IL-1β. The activated DC migrate to the draining lymph node. IL-1β is decreased in *Mpo^-/-^* mice resulting in a reduction in the number of activated DC. **(B)** Once in the draining lymph node, DC interact with naïve T cells (T) and present Ox *via* MHC class I and II to activate antigen-specific CD4^+^ and CD8^+^ T cells, respectively. Due to a reduction of activated DC reaching the draining lymph node in *Mpo^-/-^* mice, T cell priming is reduced. **(C)** For the elicitation of CHS, Ox is applied to the ears, which results in the migration of effector T cells and neutrophils into the ears. Neutrophils release MPO **(C)**, which catalyzes the production of free radicals and oxidants that drive vascular permeability leading to swelling as indicated by an increase in the thickness of the ears **(D)**. **(D)** In the absence of MPO, the production of free radicals and oxidants is reduced thereby reducing vascular permeability and ear swelling. **(A–D)** Treatment with KYC by inhibiting MPO activity would result in similar biological outcomes as those observed in *Mpo^-/-^* mice.

In this study, we investigated whether neutrophil-derived MPO plays a role in the induction and/or elicitation phases of ACD using the OX CHS mouse model. Here, we demonstrated that CHS was attenuated when MPO was deficient or inhibited and demonstrated that MPO plays a role during both the induction and elicitation phases of CHS. In the induction phase, we showed that MPO was important for optimal migration of DC from the skin to the draining lymph node where they primed naïve T cells to the OX hapten. In the elicitation phase, we found that MPO was essential for vascular permeability leading to ear swelling. Our findings are outlined in graphic form in **Figure 7**. These cumulative data suggest that neutrophils are a mediator of ACD through MPO release.

Although MPO is well characterized as a microbiocidal (36), it is now known to be pathogenic in a variety of diseases. Activation of neutrophils and subsequent MPO release also occurs in response to endogenous inflammatory stimuli even in the absence of infection (37), which is consistent with our previous studies demonstrating a pathogenic role for MPO in mouse models of multiple sclerosis, stroke and sickle cell disease (25, 26, 38). The exact mechanisms whereby MPO is pathogenic is not well understood (15, 36, 37, 39, 40). A molecular footprint of MPO are proteins containing chlorinated tyrosine (Cl-Tyr), which along with an additional MPO halogenation product 3-bromotyrosine, were reduced in MPO-deficient mice with sepsis (41). MPO-derived products not only induce oxidative stress but have also been shown to increase pro-inflammatory cytokine production (18, 42). Of particular interest is IL-1β, whose production by keratinocytes in CHS was shown to promote DC emigration from skin to the draining lymph node (7–9). In addition, IL-1β was shown to be rapidly upregulated in Langerhans cells following hapten application (43). The finding that antibody neutralization of IL-1β prior to sensitization abrogated CHS provides evidence of its pathogenic role (44). This was further supported by an impairment in CHS in mice deficient in inflammasome components required to generate active IL-1β (45). Our finding that *Mpo^-/-^* mice with CHS failed to upregulate IL-1β in the skin provides strong evidence that MPO is a critical factor promoting IL-1β transcription and/or activation by the inflammasome (46).

We showed that the absolute number and percentage of migratory DC was reduced in *Mpo^-/-^* mice with CHS, which is consistent with studies showing that neutrophils activate and induce DC migration to local lymph nodes during microbial infection, neuro-inflammation, as well as CHS resulting in activated T cell production of IL-17 and IFN-γ (12, 47–50). While these studies did not investigate MPO as a mechanism, others showed that MPO was measurable in the draining lymph node several hours after OVA/LPS injection that was correlated to a reduction in the expression of the lymph node homing receptor CCR7 on DC (17). However, the former study used LPS and not a hapten and did not focus on cutaneous DC and their migratory potential. It is well known that epidermis and dermis contain differential populations of antigen presenting cells. Langerhans cells are found in the epidermis, while the dermis contains two populations of DC with variable abilities to exhibit activation or inhibitory functions in CHS (51), which implies that MPO could have a differential impact on DC function depending on their phenotype and location.

Following hapten application to the skin, particularly Langerhans cells, become activated and emigrate to the draining lymph node, where they activate antigen-specific T cells (8, 52). Thus, the direct consequence of reduced DC migration to the draining lymph node would be reduced numbers of antigen presenting cells and abrogated T cell priming. Our findings of reduced numbers of CD4 and CD8 Teff cells in *Mpo^-/-^* mice with CHS as compared to WT are consistent with the reduced numbers of migratory DC. Of interest is that the nature of the immune response was not altered in *Mpo^-/-^* mice as shown by no difference in the percentage of CD4 and CD8 T cell undergoing proliferation or producing IFN-γ production. In addition, while the absolute number of Treg was reduced their proportion was not, indicating direct immune suppression is not likely the mechanism for the reduced numbers of T cells in *Mpo^-/-^* mice with CHS. These data indicate that MPO inhibition could be used to modulate the extent of the adaptive immune response without compromising its protective functions.

Our finding that adoptive transfer of WT neutrophils into MPO-deficient mice resulted in a significantly attenuated disease is consistent with other studies showing a crucial role for neutrophils during elicitation. The CHS response was shown to be inhibited by the depletion of neutrophils prior to CHS elicitation (12, 53). Similarly, the adoptive transfer of WT effector cells to neutrophil-depleted or neutrophil deficient animals abrogated CHS ear swelling (12). Ear swelling is associated with an increase in vascular permeability, which was shown to be mediated by neutrophils in many pathological conditions such as neurological diseases, sepsis and ischemia-reperfusion (54, 55). We also showed that MPO is essential for vascular permeability induced during the CHS elicitation phase. ECIS *in vitro* studies provided evidence that MPO alone is sufficient to induce vascular permeability, which is consistent with previous findings (35). While these cumulative findings indicate that neutrophil-derived MPO is a potent inducer of vascular permeability, the precise mechanism(s) evoked *in vivo* is not known.

## Conclusion

In this study, we provide evidence that MPO is an important neutrophil effector molecule in CHS. We found that during hapten sensitization MPO is required for both optimal IL-1β production in the skin and DC activation and emigration to the draining lymph node thereby regulating the magnitude of the adaptive immune response. We also found a role for MPO in the elicitation phase in which it plays an essential role in induction of vascular permeability. These cumulative findings indicate the MPO is a potential therapeutic target for the treatment of persistent ACD in humans. Given that MPO is a potent bactericidal (36), inhibition of MPO systemically may lead to an increase in infections. However, several studies have shown that the majority of MPO-insufficient humans are asymptomatic, although the risk of severe infections was higher (56–58). A benefit of inhibiting MPO with the tripeptide KYC or a similar drug is that its effects would be quickly reversed upon drug withdrawal.

## Data Availability Statement

The raw data supporting the conclusions of this article will be made available by the authors, without undue reservation.

## Ethics Statement

The animal study was reviewed and approved by Institutional Animal Care and Use Committee of the Medical College of Wisconsin.

## Author Contributions

Conceptualization: AS, MS, KP, and BD. Investigation: AS, CG, and LD. Formal analysis: AS and BD. Funding acquisition: BD. Resources: BD. Writing—original draft: AS and BD. Writing—review and editing: AS, CG, LD, MS, KP, and BD. All authors contributed to the article and approved the submitted version.

## Funding

This work was supported in part by National Institutes of Health grants 1R56AI129348 (BD) and 1R56AI122655 (BD), the National Multiple Sclerosis Society grant RG 4975A1/2 (BD), the Versiti Blood Research Foundation and the Gallagher Fellowship Award at the Blood Research Institute of Versiti Wisconsin (AS).

## Conflict of Interest

KP is a founder, CSO, and owner of ReNeuroGen LLC, a small pharmaceutical company whose goal is to develop KYC to treat sickle cell disease and multiple sclerosis.

The remaining authors declare that the research was conducted in the absence of any commercial or financial relationships that could be construed as a potential conflict of interest.
