# Supplementary material for: Neutrophil-Derived Myeloperoxidase Facilitates Both the Induction and Elicitation Phases of Contact Hypersensitivity
Source: Front Immunol. 2021 Jan 25;11:608871. doi: 10.3389/fimmu.2020.608871 (PMC7868335; doi:10.3389/fimmu.2020.608871)
Supplement: Supplementary file 1 [file Table_1.pdf]

Supplementary Table 1. Anti-mouse Antibodies Utilized

Specificity	Conjugation	Clone	Commercial source[a,b]
CD4	FITC	GK1.5	BioLegend
CD4	BV421	GK1.5	BioLegend
CD4	APC-efluor780	RM-4.5	ThermoFisher
CD8	Pacific Blue	53-6.7	BioLegend
CD8	PeCy5	53-6.7	BioLegend
CD8	efluor450	53-6.7	ThermoFisher
CD11b	efluor450	M1/70	ThermoFisher
CD11c	APC-Cy7	N418	BioLegend
CD25	BV605	PC61	BioLegend
CD62L	APC	MEL-14	BioLegend
B220	AF594	RA3-6B2	BioLegend
TCRβ	FITC	H57-597	BioLegend
TCRβ	PE-Cy5	H57-597	ThermoFisher
MHC class II	FITC	AF6-120.1	BioLegend
MHC class II	APC	M5/114.15.2	BioLegend
Ly6G	APC-Cy7	1A8	BioLegend
Ly6C	APC	HK1.4	BioLegend
Ly6G	biotin	1A8	BioLegend
Gr1	AF647	RB6-8C5	BioLegend
IFN-γ	APC	XMG1.2	BioLegend
IL-17A	PE	TC11-18H10.1	BioLegend
Foxp3	PE	FJK-6s	ThermoFisher

[a]BioLegend, San Diego, CA
[b]ThermoFisher Scientific, Waltham, MA
